# Supplementary material for: Modeling Thoracic Aortic Dissection Using Patient‐Specific iPSCs Reveals VSMC Dysfunction and Extracellular Matrix Dysregulation
Source: Stem Cells Int. 2025 Dec 19;2025:4700736. doi: 10.1155/sci/4700736 (PMC12767432; doi:10.1155/sci/4700736)
Supplement: Supplementary file 1 — Supporting Information Figure S1: The iPSC reprograming process with morphology changes at different stages from primary VSMC to established iPSC colonies. Scale bar = 250 µm. Figure S2: The cell morphology changes during mesoderm‐mediated SMC differentiation from iPSCs. Scale bar = 250 µm. [file SCI-2025-4700736-s001.docx]

**Supplementary materials**

**
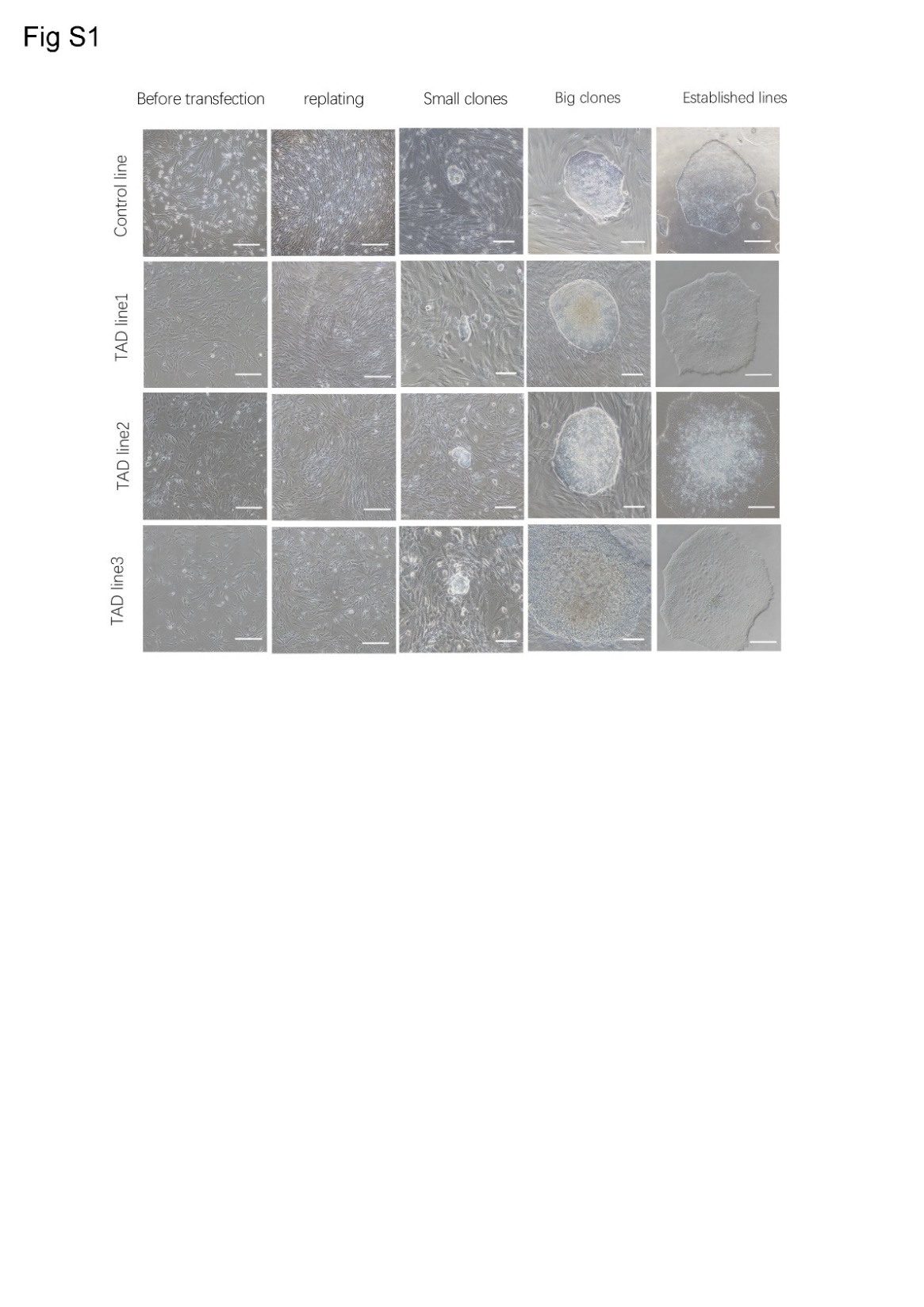
Fig.S1**  **The iPSC** **reprogramming process with morphology changes at different stages from primary VSMC to established iPSC colonies. Scale bar=250μm.**

Before reprogramming, cells exhibited a spindle-shaped morphology. Following reprogramming, an increase in cell proliferation was observed. Approximately 15 days later, small colonies began to form, and an induced pluripotent stem cell (iPSC) line was successfully established around 25 days post-electroporation.

**
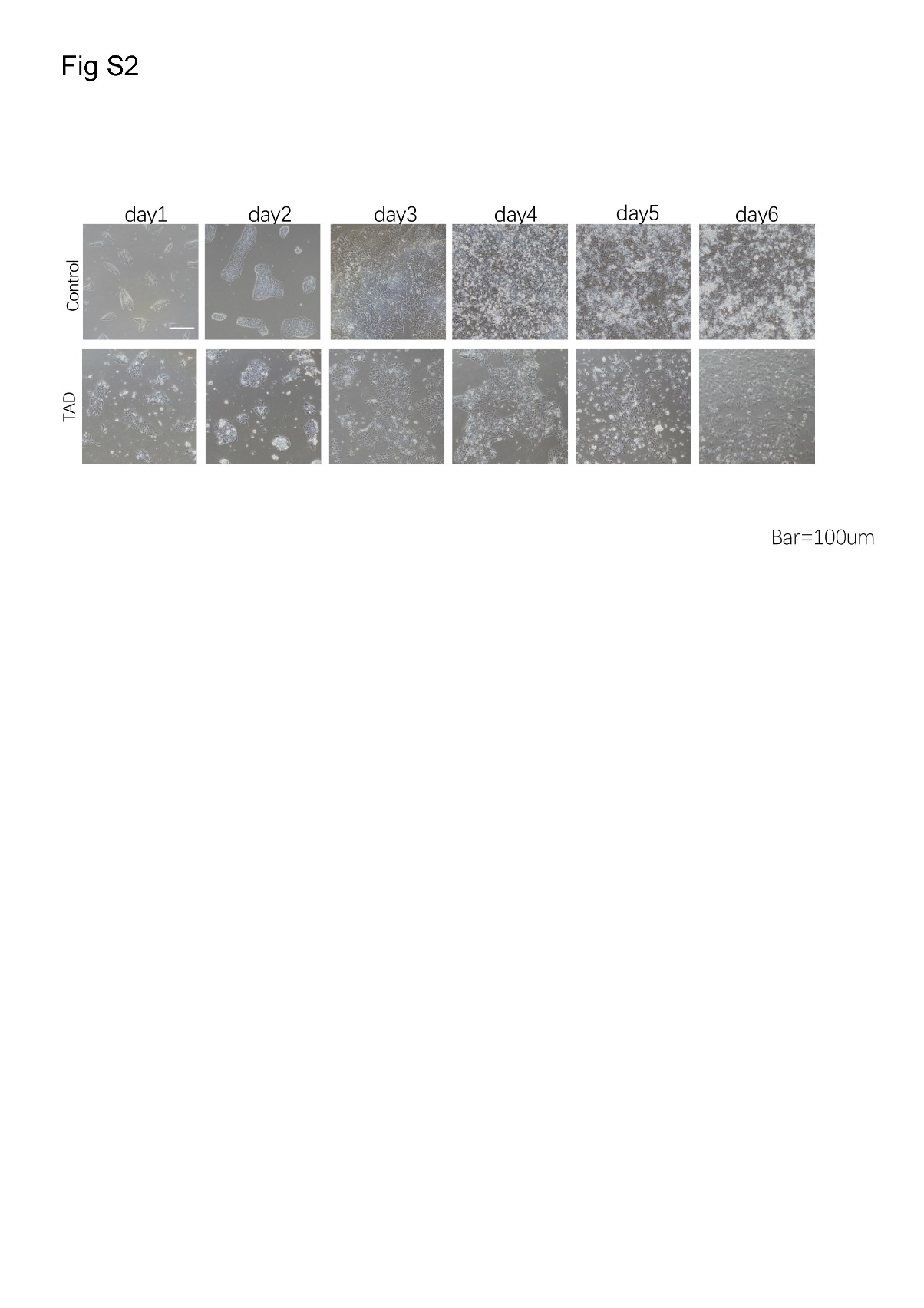
**

**Fig.S2** **The cell morphology changes during mesoderm-mediated SMC differentiation from iPSCs. Scale bar=250μm.**

day1-day3, the cellular morphology underwent a transition from a pluripotent stem cell state to polygonal mesodermal progenitors. day4-day7, these mesoderm-derived cells progressively differentiated into smooth muscle cells
